# Recruiting people with severe mental illness through community pharmacies: real-world experiences from a UK study

**DOI:** 10.1186/s12875-020-01243-5

**Published:** 2020-08-20

**Authors:** Hannah Macfarlane, Ian Maidment

**Affiliations:** 1 School of Life and Health Sciences, Aston University, Birmingham, B4 7ET UK; 2 Pharmacy Department, Secure and Complex Care, Birmingham and Solihull Mental Health NHS Foundation Trust, Unit 1, B1, 50 Summer Hill Road, Birmingham, B1 3RB UK

**Keywords:** Severe mental illness, Recruitment, Research methods, Community pharmacy

## Abstract

**Background:**

Proxy recruitment of patient participants through community pharmacies may be a valuable strategy to maximise participation. This paper focuses on the feasibility of such a recruitment strategy for research involving people who experience severe mental illness.

**Methods:**

Fifty-three community pharmacies, including 50 ‘Research Ready’ pharmacies, were asked to recruit people with severe mental illness for participation in research. Pharmacists were asked to provide participant information to anyone presenting a prescription meeting specific criteria.

**Results:**

Thirteen recruitment sites (25%) (from 4 distinct organisations) were approved to recruit patient participants. Eighty-five percent (*n* = 11) failed to recruit any potential participants.

**Conclusions:**

Proxy recruitment of people with severe mental illness through community pharmacies was challenging with challenges in both pharmacy- and participant-recruitment. Further investigation into supporting community pharmacists’ engagement with recruiting patients with SMI as research participants is required.

## Background

The importance of recruiting a diverse sample of research participants must be balanced against the practical limitations imposed by limited resources. In an attempt to reach a balance between these two issues, the possibility of recruitment activity being undertaken by people outside of the immediate research team, such as by community pharmacies (CPs), can be considered [1–3].

People who experience mental illness, or their carers, are in regular contact with pharmacies which might present a suitable option as a proxy recruitment site. Previous research has reported on the feasibility of proxy recruitment of people with a chronic health condition through CPs [1].

Here, the pharmacy and participant recruitment challenges experienced in a UK-based study are reported. This qualitative study involving semi-structured interviews aimed to determine participants’ perceptions of the roles of community pharmacists in supporting medicines optimisation for people who experience mental illness. This work is part of a programme of postgraduate research looking at how pharmacists work with people with mental illness.

## Methods

Potential recruitment sites were initially sought from a list of Royal Pharmaceutical Society (RPS)-accredited Research Ready Pharmacies (RRPs), accessed through the National Institute for Health Research Clinical Research Network: West Midlands (NIHR CRN:WM). The RRP programme is an “online self-accreditation tool covering the basic requirements for undertaking primary care research in the UK” [4].

Fifty NIHR CRN:WM RRPs received study information from CRN:WM gatekeeper pharmacists. Due to poor RRP recruitment, we subsequently expanded to pharmacies with which we had professional contacts. Three additional pharmacies were subsequently contacted this way and site visits were offered to all 53 pharmacies.

Pharmacists were asked to identify potential patient participants based on prescribed medicines. No specific guidance was provided regarding how pharmacists should introduce the study to potential patient participants. Following expression of interest, potential patient participants were contacted by the lead researcher to discuss the study and confirm participation.

No incentive was offered to pharmacies, but a £10 gift voucher plus expenses was offered to patient participants. Three months were originally allowed for the recruitment of research participants.

## Results

### Pharmacy recruitment

Research Ready Pharmacies received study information via email, phone or site visit. Where no response was received, at least one further contact was made. Four expressions of interest resulted from three independent pharmacies and a chain of 10 premises (on behalf of which interest was expressed by a single employee responsible for the chain’s involvement in research).

The lead researcher met with pharmacists from two of the three independent pharmacies to discuss the study and contacted the third by telephone. A meeting was also held with a single representative for the chain of pharmacies. Following this, recruitment site approval was granted for one of the independent pharmacies and each premises in the chain. The remaining two independent premises withdrew from contact. Twenty two per cent (11/50) of CRN:WM-linked RRPs participated in the study; falling to 4% (2/50) if the chain of pharmacies for whom expression of interest was received from a single individual, is considered a single entity.

Following extension to the ethical approval, study information was distributed to three further pharmacies via professional contacts. Two were subsequently granted site approval. Finally, study details were sent to the Birmingham and Solihull Local Pharmaceutical Committee for distribution to members but no expressions of interest by pharmacies were received.

The mean time taken for site approval for each recruiting pharmacy (*n* = 13) from the date of ethics approval was 62 days (range 31–102).

### Participant recruitment

Six potential patient participants expressed interest in study participation arising from two pharmacies; one RRP and one pharmacy approached via professional contacts. Eleven sites (85%) failed to recruit any potential patient participants. Email contact was maintained throughout the recruitment phase, with repeated offers of support or face-to-face meetings. Recruitment was closed after 12 months.

Patient retention rate from expression of interest to interview completion was 50% (*n* = 3). Expressions of interest from patients were received on average 21 days (range 7–31, *n* = 6) following site approval and average time to interview completion was 49 days (range 31–62, *n* = 3).

## Discussion

Recruiting patients with mental illness through CPs was reliant on the willingness and capacity of pharmacists to support the research. In this study, this strategy was ineffective, despite literature support for the method [1, 2, 5]. The local RRP network was of limited value in terms of recruitment and patient recruitment was curtailed after 12 months; four times the planned duration. Where RRP networks are used in future, simultaneous employment of additional strategies such as use of professional contacts and snowballing may enhance recruitment.

Personal contact between the research team and the recruitment site may be beneficial in terms of the commitment subsequently demonstrated by that site [6]. A limited number of pharmacies accepted our offers of a personal visit. Although these appeared well received, they did not correlate with subsequent recruitment success.

Pharmacies may have struggled to identify potential patient participants. Initial meetings with pharmacists were promising, with pharmacists suggesting that they could easily identify potential participants. However, 85% (11/13) were unable to recruit any patients. Two pharmacists fed back that they had not identified anyone fitting the inclusion criteria over the 12-month recruitment period.

Research has highlighted ambivalence or even negative views amongst community pharmacists regarding their willingness to prioritise research during working hours [5]. This may be exacerbated by a lack of confidence in their knowledge and skills in working with people with mental illness [7–9] or conscious or unconscious stigmatised views of people with mental illness [10]. Such stigma may affect the ability of pharmacists to build relationships with people with SMI, making it less likely that they would approach them for recruitment to research. These two factors together may create a barrier to research and, perhaps more importantly, to community pharmacy-led mental health clinical and supply services. Improvement to recruitment efficiency is critical but may not be plausible in the way described here until the community pharmacy workforce feels better equipped to develop meaningful relationships with people experiencing SMI.

Having the researcher physically present in community pharmacies might have helped to overcome some of these potential barriers by obviating the need for proxy recruitment [11] and because they would be ideally placed to promote the study. Such direct recruitment in a Danish study within pharmacies resulted in high participant recruitment (108 interviews), but used 58 pharmacy interns as the researchers to achieve this [12]. Amending the recruitment strategy in this way has clear resource implications.

Reluctance to refer people for inclusion in research has been attributed to lack of confidence and skill as well as misconceptions about the research [13]. Additionally, community pharmacists may have overestimated their capacity for engagement in research [4] or misunderstood the selection criteria thus reducing the number of potential participants approached [2]. Such factors may have contributed to the poor recruitment. Therefore, offering education and training to clinicians may benefit study recruitment [6]. With greater resources, it might have been possible to bolster pharmacy recruitment by arranging a study launch meeting for community pharmacists, personal visits to each potential recruitment site, offering brief training on the treatment of SMI or the payment of incentives to the pharmacy for each completed interview [1].

### Limitations

The study described here was limited to a single geographical region in the UK.

Since the aim of the underpinning research was different to that of this report, some data which may be of value are not available as they were not collected. For example, the number of recruitment packs handed out and the previous research experience of the pharmacies. Recruitment might have been enhanced had pharmacy technicians been included in the method but this was not addressed in this study. Finally, the barriers to recruitment might have been better elucidated by collecting qualitative data from pharmacies who chose not to engage or who were unable to recruit any participants, and from patients who did not participate.

## Conclusions

This study highlights potential difficulty in recruiting research participants with severe mental illness by proxy through their community pharmacies. Researchers considering proxy recruitment strategies for their studies are encouraged to consider the difficulties described here. For proxy recruitment strategies to be effective, a personal relationship between the researcher and the recruiters with ongoing personal contact between the two parties appears vital. Future research should focus on how community pharmacists can be better supported to engage with research as part of their day-to-day practice and on supporting them to work with people with SMI in the research context.

## Data Availability

Not applicable.

## Abbreviations

CPs: Community pharmacies
CRN: Clinical research network
NHS: National health service
NIHR: National institute for health research
RPS: Royal pharmaceutical society
SMI: Severe mental illness
